# Metabolic dysfunction-associated fatty liver disease is an early predictor for testosterone deficiency in aging men without metabolic syndrome

**DOI:** 10.3389/fendo.2023.1252774

**Published:** 2023-10-03

**Authors:** Chia-Chu Liu, Shu-Pin Huang, Yung-Chin Lee, Cheng-Hsueh Lee, Tsung-Yi Huang, Jiun-Hung Geng, Che-Wei Chang, Chung-Yu Lin, Yung-Shun Juan, Wen-Jeng Wu, Tusty-Jiuan Hsieh

**Affiliations:** ^1^ Department of Urology, Kaohsiung Medical University Hospital, Kaohsiung Medical University, Kaohsiung City, Taiwan; ^2^ Department of Urology, School of Medicine, College of Medicine, Kaohsiung Medical University, Kaohsiung City, Taiwan; ^3^ Department of Urology, Pingtung Hospital, Ministry of Health and Welfare, Pingtung City, Taiwan; ^4^ Research Center for Precision Environmental Medicine, Kaohsiung Medical University, Kaohsiung City, Taiwan; ^5^ Department of Urology, Kaohsiung Municipal Siaogang Hospital, Kaohsiung City, Taiwan; ^6^ Graduate Institute of Medicine, College of Medicine, Kaohsiung Medical University, Kaohsiung City, Taiwan

**Keywords:** metabolic dysfunction-associated fatty liver disease, testosterone deficiency, aging men, metabolic syndrome, insulin resistance

## Abstract

**Background and aims:**

Metabolic dysfunction-associated fatty liver disease (MAFLD) has emerged as a valuable marker for identifying individuals at increased risk of metabolic dysfunction, liver-related complications, and cardiovascular disease. However, the association between MAFLD and testosterone deficiency (TD) in aging men remains poorly understood. This study aimed to investigate the association between MAFLD and the risk of TD in aging Taiwanese men, with a specific focus on those without metabolic syndrome (MetS).

**Methods:**

A free health screening program was conducted for Taiwanese men aged over 40 years in Kaohsiung, Taiwan. Participants underwent physical examinations, completed questionnaires regarding demographics, medical history, and clinical symptoms of TD, and provided 20-mL whole blood samples for biochemical, adipocytokine, and hormonal evaluations. Fatty liver index was used to evaluate the risk of fatty liver. Diagnostic criteria for MAFLD included fatty liver along with overweight/obesity, type 2 diabetes, or evidence of metabolic dysregulation.

**Results:**

A total of 631 men (mean age: 54.4 ± 8.4 years) were enrolled. The prevalence rates of TD and MetS were significantly higher in men with MAFLD compared to those without (both p < 0.001). Additionally, the presence of MAFLD showed a significant correlation with adipocytokines associated with insulin resistance, such as adiponectin, leptin, and retinol-binding protein-4 (RBP-4) levels (all p < 0.001). Among men without MetS, those with MAFLD had a 3.89- and 4.74-fold higher risk of total testosterone < 300 ng/dL and TD, respectively, after adjusting for potential covariates.

**Conclusion:**

MAFLD is associated with an elevated risk of TD in aging Taiwanese men, particularly in the absence of MetS. This finding suggests that MAFLD could serve as an early predictor of TD, facilitating the identification of high-risk individuals and enabling timely interventions. Further research is needed to validate these findings and explore the underlying mechanisms linking MAFLD, TD, and MetS in diverse populations.

## 1 Introduction

Testosterone (T) functions as the principal sex hormone in males, playing a crucial role in both androgenic and anabolic processes. Its production reaches peak during men’s adolescence and early adulthood, but gradually declines at a rate of approximately 0.4-2.6% per year after the age of 40 (1). Reduced T levels have been associated with various clinical symptoms, including decreased libido, alterations in erectile function, decreased energy levels, diminished motivation, impaired work and physical performance, depressive symptoms, irritability, impaired concentration, and disrupted sleep patterns, all of which significantly impact men’s overall quality of life (1, 2). In men, T levels were also found to significantly associate with body fat distributions (3). Furthermore, low T has been established as a significant risk factor for cardiovascular diseases, with an observed correlation to increased incidences of stroke, myocardial infarction, cardiovascular-related mortality, and all-cause mortality among male individuals (4, 5). Testosterone deficiency (TD) is currently defined as a clinical syndrome characterized by low serum T concentrations, accompanied by a specific constellation of clinical symptoms and signs (6). Given the profound negative impact of TD on male sexuality, reproductive function, general health, and overall quality of life, it is imperative to identify reliable early predictors that can aid medical practitioners in identifying high-risk individuals and facilitating timely preventive interventions and treatment in clinical settings.

Non-alcoholic fatty liver disease (NAFLD) stands as the predominant cause of chronic liver disease, affecting approximately 25% of the adult population globally (7). Its escalating prevalence has rendered it the fastest growing cause of liver-related mortality worldwide and emerged as a significant contributor to end-stage liver disease, primary liver cancer, and the need for liver transplantation, consequently imposing a considerable burden on healthcare systems (8). NAFLD exhibits a robust and bidirectional relationship with various components of metabolic syndrome (MetS) (8–10). However, the conventional definition of NAFLD has several inherent limitations, including its inability to capture the disease’s heterogeneity, exclusionary diagnostic patterns associated with the definition, and the stigmatization resulting from its nomenclature (11). In 2020, an international panel of experts proposed a novel nomenclature, namely metabolic dysfunction-associated fatty liver disease (MAFLD), aiming to emphasize the significant contribution of metabolic risk factors to the onset and progression of liver disease (12, 13). The diagnostic criteria for MAFLD encompass the presence of hepatic steatosis in conjunction with one of three features: overweight or obesity, type 2 diabetes, or being lean or of normal weight with evidence of metabolic dysregulation (12, 13). This shift from NAFLD to MAFLD represents a crucial milestone in moving towards a more comprehensive, equitable, and patient-centered approach in addressing the profound challenges associated with this disease (12, 13).

While extensive research has been conducted on the association between MetS and TD, the relationship between NAFLD and TD has only recently gained attention (14, 15). Although NAFLD is commonly acknowledged as the hepatic manifestation of MetS, emerging evidence suggests that it may serve as a precursor to the subsequent development of MetS (16, 17). However, there is a paucity of literature investigating the correlation between MAFLD, the newly established comprehensive definition of fatty liver, and TD in aging men (18). Additionally, no studies have been undertaken to evaluate the potential of MAFLD in predicting the risk of TD in individuals without MetS. Hence, the objective of this study is to assess the association between the risk of MAFLD and TD in an aging Taiwanese male population, with a particular focus on those without MetS.

## 2 Materials and methods

### 2.1 Subjects and study protocol

The present study utilized cross-sectional data obtained from Taiwanese males aged 40 years and above, which were collected during a free health screening program conducted by a medical center in Kaohsiung City, Taiwan, between June 2012 and August 2014. Subject selection criteria were implemented following previously described methods (see **Supplementary Table S1**) (1, 19–21). The exclusion criteria included the following: (1) individuals with a documented history of significant psychiatric disorders, substance abuse disorders, advanced liver and/or renal disease, or malignancy; (2) individuals who were currently utilizing hormone therapy, undergoing anti-androgen treatment, consuming steroidal agents, or taking antifungal drugs; and (3) individuals who had incomplete evaluations during the study period (1, 19–21). The study followed the ethical guidelines stated in the Declaration of Helsinki and obtained approval from the Institutional Research Ethics Committee of the Kaohsiung Medical University Hospital. Written informed consent was obtained from all participants involved in the study.

Trained researchers conducted interviews with participants using a structured questionnaire to gather demographic information, detailed medical, surgical, and psychosexual history, current medication usage, and lifestyle data (1, 19–21). Additionally, the androgen deficiency in aging male (ADAM) questionnaire was employed to evaluate the clinical symptoms of TD in each participant. A participant was considered positive for symptoms suggestive of TD if they reported decreased libido or strength of erection, or if they responded positively to any three of the non-specific questions, including fatigability, mood change, loss of height, and decreased muscle strength (1, 20–22). Participants were classified as alcohol drinkers, cigarette smokers or betel quid chewers if they had regularly consumed any alcoholic beverage ≥1 times per week, had smoked ≥10 cigarettes per week, or had chewed ≥7 betel quids per week, respectively, for at least 6 months. Those who were still using any of the aforementioned substances within one year before the interview were classified as current users (1, 20, 21, 23). Regular exercise was defined as participating in aerobic exercise at least once a week for a minimum duration of 20 minutes, leading to perspiration, within one year prior to the interview (19–21).

### 2.2 Physical measurements

Each participant underwent physical measurements, which included recording their blood pressure, body weight (in kilograms), height (in centimeters), and waist circumference (WC). Blood pressure was measured twice, following a resting period of 15 minutes or more, using a mercury sphygmomanometer, and the average values were documented for subsequent analysis. WC was measured at the midpoint between the inferior costal margin and the superior border of the iliac crest along the mid-axillary line. Body mass index (BMI) was calculated by dividing the body weight (in kilograms) by the square of the body height (in meters).

### 2.3 Biochemical, hormonal and adipocytokine analyses

Following an overnight fasting period of 8 hours, all participants underwent a blood draw of 20 cc between 8:00 and 11:00 AM on the day of screening for the analysis of various parameters, including routine biochemical profiles, lipid panels, serum glucose, adipocytokines, and hormone levels. All analytical procedures adhered to previously established protocols (1, 19–21). Specifically, serum total testosterone (TT) and sex hormone-binding globulin (SHBG) levels were determined using a DPC Immulite analyzer (Diamond Diagnostics, Holliston, MA), with inter-assay coefficients of variation (CV) of 8.4% and 4.8%, respectively, and intra-assay CV of 5.2% and 3.5%, respectively (1, 19–21). Serum adiponectin and leptin levels were measured using Millipore’s RIA kits (Missouri, USA), with intra-assay CV ranging from 3.4% to 8.3% for adiponectin and 1.78% to 6.21% for leptin, and inter-assay CV ranging from 3.0% to 6.2% for adiponectin and 6.90% to 9.25% for leptin. Serum retinol binding protein-4 level (RBP-4) was measured using Plasma’s enzyme-linked immunosorbent assay kit (TX, USA), with intra-assay CV below 10.0% and inter-assay CV below 12.0% (19, 21, 24).

### 2.4 Definition of TD and MetS

The diagnosis of TD was based on the guidelines provided by the American Urological Association (AUA) for the evaluation and management of TD (6). TD was defined as having both a TT level below 300 ng/dL and positive clinical symptoms determined through the ADAM questionnaire (20, 21). An individual was classified as having MetS if they met at least three of the following five criteria: (1) WC ≥ 90cm; (2) high density lipoprotein (HDL) cholesterol < 40 mg/dL or lipid medication use; (3) triglyceride (TG) ≥ 150 mg/dL or use of lipid−lowering therapy; (4) blood pressure (BP) ≥ 130/85 mm Hg or use of antihypertensive medication; (5) fasting blood glucose (FBG) ≥ 100 mg/dL or diagnosed as having type 2 diabetes, following modified criteria derived from the National Cholesterol Treatment Adult Treatment Panel III (NCEP ATP-III) definitions proposed by the Bureau of Health Promotion in Taiwan (19–21).

### 2.5 The calculation of fatty liver index and definition of MAFLD

The FLI was calculated using the formula established by Bedogni et al: FLI = (e 0.953*loge (TG) + 0.139*BMI + 0.718*loge (gamma-glutamyltransferase (GGT)) + 0.053*WC-15.745)/(1 + e 0.953*loge (TG) + 0.139*BMI + 0.718*loge (GGT) +0.053*WC-15.745)*100 (25). The risk of fatty liver in Taiwanese men was classified based on the criteria proposed by Yang et al. A FLI value of 35 or greater was used to indicate the presence of fatty liver (21, 26).

The diagnostic criteria for MAFLD included the presence of fatty liver (FLI ≥ 35) along with one of the following three features: overweight or obesity, type 2 diabetes, or lean or normal weight with evidence of metabolic dysregulation, as outlined in the international expert consensus statement from 2020 (12, 13). Metabolic dysregulation was defined as the presence of at least two of the following metabolic risk abnormalities: (1)WC ≥ 90 cm; (2) BP ≥ 130/85 mm Hg or the use of antihypertensive medication; (3) TG ≥ 150 mg/dL or the use of lipid-lowering therapy; (4) HDL cholesterol levels < 40 mg/dL or the use of lipid medication; (5) Prediabetes (FBG: 100–125 mg/dL or hemoglobin A1C: 5.7%-6.4%) (12, 13).

### 2.6 Statistical analysis

Quantitative data were expressed as mean ± standard deviation (SD), while categorical data were presented as numbers (n) and percentages. The chi-square test or Fisher’s exact test was employed to compare qualitative variables, whereas the Student’s t-test or one-way analysis of variance (ANOVA) was used to compare quantitative variables. Multivariate regression analyses were performed to examine the relationships between the presence of MAFLD and the risks of having TT levels below 300 ng/dL and TD. Adjustments were made for potential covariates. To ensure the robustness of the analyses, sensitivity tests were conducted, comparing the results using the original criteria for fatty liver risk proposed by Bedogni et al., which defined FLI ≥ 60 as indicative of fatty liver (25). All statistical analyses were carried out using SPSS version 18.0 (SPSS Inc., Chicago, IL, USA).

## 3 Results

Out of the initial 667 men who participated in the health screening, a total of 36 subjects were excluded from the analysis. The reasons for exclusion were current malignancies (15 cases), current use of medications that could interfere with the measurement of natural T levels (10 cases), and incomplete evaluation (11 cases). As a result, the final study population consisted of 631 subjects, with a mean age of 54.4 ± 8.4 years (range: 40-80 years). The baseline characteristics of the study population are summarized in **Table 1**. Among the participants, 78 (12.4%) were diagnosed with MetS, 229 (36.3%) with MAFLD, and 70 (11.1%) with TD. The mean value of TT level was 411.7 ± 121.4 ng/dl (range: 120.0-1230.0 ng/dl) and FLI was 32.2 ± 22.4 (range: 11.0-99.9).

**Table 1:** Baseline characteristics of study population (N =631).

Characteristic	N (%)	Mean±SD	Median (IQR)	Range
Age (years)		54.4±8.4	55.0 (52.0-58.0)	40-80
Age groups				
40-49 years	95 (15.1)			
50-59 years	407 (64.5)			
60-69 years	114 (18.1)			
≧70 years	15 (2.4)			
Education status				
Primary school or less	20 (3.2)			
Secondary/high school	279 (44.2)			
At least some college	332 (52.6)			
Waist circumference (cm)		84.2±9.0	84.0 (79.0-89.0)	57-120
BMI		25.1±3.0	25.1 (23.1-26.8)	16.5-40.6
Normal weight	222 (35.2)			
Overwight	271 (42.9)			
Obesity	138 (21.9)			
Hypertension	146 (23.1)			
Diabetes	55 (8.7)			
Current smoking	107 (17.0)			
Current alcohol drinking	90 (14.3)			
Current Betel quid chewing	8 (1.3)			
Regular exercise	485 (76.9)			
Metabolic syndrome	78 (12.4)			
The number of MetS components		1.4±1.1	1.0 (1.0-2.0)	0-5.0
Fatty liver index		32.2±22.4	25.9 (14.3-46.4)	11.0-99.9
Fatty liver index≧35	234 (37.1)			
MAFLD	229 (36.3)			
Total testosterone<300 ng/dl	82 (13.0)			
Testosterone deficiency	70 (11.1)			
Laboratory Data				
Total testosterone (ng/dl)		411.7±121.4	394.0 (348.0-462.0)	120-1230
SHBG (nmol/L)		38.6±16.4	37.0 (27.8-47.4)	9.0-144.0
Albumin (g/dl)		4.4±0.3	4.4 (4.3-4.5)	3.6-9.6
Fasting blood glucose (mg/dl)		101.7±26.8	95.0 (89.0-103.0)	42.0-349.0
Triglyceride (mg/dl)		118.0±64.8	101.0 (74.0-140.0)	32.0-485.0
Total Cholesterol (mg/dl)		200.1±36.7	201.0 (176.0-223.0)	68.0-351.0
HDL (mg/dl)		49.5±9.9	48.5 (43.1-54.8)	22.5-89.4
Adiponectin (μg/ml)		4.3±1.9	4.0 (3.0-5.1)	1.0-13.2
leptin (ng/ml)		3.6±2.7	2.2 (1.7-5.8)	0.5-11.3
RBP-4 (μg/ml)		25.0±5.3	24.9 (21.6-27.7)	0.4-42.3

SD, standard deviation; IQR, interquartile range; BMI, body mass index; MetS, metabolic syndrome; MAFLD, metabolic dysfunction associated fatty liver disease; SHBG, sex hormone-binding globulin; HDL, high-density lipoprotein; RBP-4, retinol binding protein-4.

### 3.1 Clinical characteristics and laboratory data between subjects with and without MAFLD

A comparison of clinical characteristics and laboratory data was performed between subjects with and without MAFLD, as shown in **Table 2**. Subjects with MAFLD exhibited significantly lower education status (p=0.011) and a lower prevalence of regular exercise (p<0.001). They also had a higher prevalence of overweight/obesity (p<0.001), hypertension (p<0.001), diabetes (p<0.001), MetS (p<0.001), TT levels below 300 ng/dL (p<0.001), TD (p<0.001), and current habits of smoking (p=0.007) and betel quid chewing (p<0.001) compared to those without MAFLD. Furthermore, subjects with MAFLD exhibited significantly higher values of FLI, serum leptin, and RBP-4 levels, but lower serum TT, SHBG and adiponectin levels, compared to those without MAFLD (all p<0.001).

**Table 2:** The comparison of clinical characteristics and laboratory data between subjects with and without MAFLD.

	Subjects with MAFLD	Subjects without MAFLD	P value
	(n=229)	(n=402)	
Age (years)	54.3±9.2	54.4±7.9	0.913
Education status, n(%)			
Primary school or less	12 (5.2)	8 (2.0)	0.011
Secondary/high school	110 (48.0)	169 (42.0)	
At least some college	107 (46.8)	225 (56.0)	
Waist circumference (cm)	91.4±7.2	80.2±6.0	<0.001
BMI	27.5±2.7	23.7±2.2	<0.001
Overweight/Obesity, n(%)	219 (95.6)	190 (47.3)	<0.001
Hypertension, n(%)	78 (34.1)	68 (16.9)	<0.001
Diabetes, n(%)	32 (14.0)	23 (5.7)	<0.001
Current smoking, n(%)	51 (23.3)	56 (13.9)	0.007
Current alcohol drinking, n(%)	38 (16.6)	52 (12.9)	0.206
Current Betel quid chewing, n(%)	8 (3.5)	0 (0)	<0.001
Regular exercise, n(%)	145 (63.3)	340 (84.6)	<0.001
Metabolic syndrome (MetS), n(%)	68 (29.7)	10 (2.5)	<0.001
the number of MetS components	2.1±1.1	0.9±0.8	<0.001
Total testosterone<300 ng/dl	49 (21.4)	33 (8.2)	<0.001
Testosterone deficiency, n(%)	44 (19.2)	26 (6.5)	<0.001
Laboratory Data			
Fatty liver index	57.3±15.8	18.0±9.0	<0.001
Total testosterone (ng/dl)	385.4±133.7	426.7±111.2	<0.001
SHBG (nmol/L)	30.9±13.7	43.0±16.1	<0.001
Albumin (g/dl)	4.4±0.2	4.4±0.3	0.78
Fasting blood glucose (mg/dl)	108.1±35.8	97.0±19.0	<0.001
Triglyceride (mg/dl)	159.3±75.5	94.4±42.7	<0.001
Total Cholesterol (mg/dl)	205.8±41.3	197.9±33.6	0.016
HDL (mg/dl)	46.0±8.6	51.5±10.0	<0.001
Adiponectin (μg/ml)	3.8±1.8	4.8±1.8	<0.001
leptin (ng/ml)	4.4±3.1	2.8±2.0	<0.001
RBP-4 (μg/ml)	26.5±5.6	24.2±5.1	<0.001

MAFLD, metabolic dysfunction associated fatty liver disease; BMI, body mass index; MetS, metabolic syndrome; SHBG, sex hormone-binding globulin; HDL, high-density lipoprotein; RBP-4, retinol binding protein-4.

### 3.2 The distribution of TT levels below 300 ng/dL and TD according to the presentation of MAFLD in participants with and without MetS

A comparative analysis was conducted to assess the distribution of TT levels below 300 ng/dL and TD based on the presence of MAFLD in individuals with and without MetS, as outlined in **Table 3**. The distribution of TT levels below 300 ng/dL and TD exhibited a significant difference only among subjects without MetS (both p<0.001), while no significant difference was observed among those with MetS. In subjects without MetS, both the prevalence of TT levels below 300 ng/dL and TD increased with the presence of MAFLD.

**Table 3:** The distribution of total testosterone<300 ng/dL and testosterone deficiency according to the presentation of MAFLD in participants with and without metabolic syndrome.

	Subjects with TT<300ng/dl	Sujbects with TT≧300 ng/dl	P value	Subjects with TD	Subjects without TD	P value
Subjects without MetS, n (%)						
MAFLD (-)	32 (8.2)	360 (91.8)	<0.001	25 (6.4)	367 (93.6)	<0.001
MAFLD (+)	36 (22.4)	125 (77.6)		35 (21.7)	126 (78.3)	
Subjects with MetS, n (%)						
MAFLD (-)	1 (10.0)	9 (90.0)	0.483	1 (10)	9 (90.0)	0.775
MAFLD (+)	13 (19.1)	55 (80.9)		9 (13.2)	59 (86.8)	

MAFLD, metabolic dysfunction associated fatty liver disease; TT, total testosterone; TD, testosterone deficiency; MetS, metabolic syndrome.

### 3.3 The associations of MAFLD with the risks of TT levels below 300 ng/dL and TD in participants without MetS

In subjects without MetS, multivariate regression analyses were performed to investigate the associations between MAFLD and the risks of having TT levels below 300 ng/dL and TD. After adjusting for confounding factors such as age, educational level, habits of cigarette smoking, betel quid chewing, alcohol drinking, and regular exercise, subjects with MAFLD had significantly increased risks of TT levels below 300 ng/dL and TD compared to those without MAFLD (both p<0.001). Specifically, subjects with MAFLD faced a 3.89-fold higher risk of having TT levels below 300 ng/dL and a 4.74-fold higher risk of having TD compared to their counterparts without MAFLD. Furthermore, sensitivity tests were conducted using the original criteria proposed by Bedogni et al. for defining fatty liver risk. The findings demonstrated a similar trend, indicating that subjects with MAFLD, according to the original criteria, had a 3.02-fold higher risk of having TT levels below 300 ng/dL and a 3.41-fold higher risk of having TD compared to those without MAFLD. Additionally, a significant negative correlation existed between MAFLD presentation and TT levels in multivariate linear regression, regardless of the chosen criteria for defining fatty liver risk (data not presented).

## 4 Discussion

In this study, we investigated the association between MAFLD and TD in a cohort of 631 Taiwanese male participants aged 40-80 years. Our analysis revealed that subjects with MAFLD had significantly higher rates of TT levels below 300 ng/dL, TD, and MetS compared to those without MAFLD (**Table 2**). Furthermore, they exhibited significantly elevated levels of serum leptin and RBP-4, along with decreased levels of serum adiponectin, which may indicate a potential link to insulin resistance (**Table 2**). The distribution of TT levels below 300 ng/dL and TD significantly differed only among subjects without MetS based on the presence of MAFLD, with an increased prevalence of both conditions (**Table 3**). Importantly, after adjusting for potential confounding factors, individuals with MAFLD faced significantly higher risks of TT levels below 300 ng/dL and TD compared to those without MAFLD (**Table 4**). These findings highlight a noteworthy association between MAFLD and TD, suggesting a potential role of MAFLD in the development of TD in individuals without MetS.

**Table 4:** Associations of MAFLD with the risks of total testosterone <300 ng/dL and testosterone deficiency in participants without metabolic syndrome.

(A)Fatty liver index≧35				
	Subjects with TT<300ng/dl	Sujbects with TT≧300 ng/dl	Crude OR	Adjuested OR^*^
	(n=68)	(n=485)	(95% CI)	(95% CI)
MAFLD (-)	32 (8.2)	360 (91.8)	Ref	Ref
MAFLD (+)	36 (22.4)	125 (77.6)	3.24 (1.93-5.44)	3.89 (2.16-7.00)
	Subjects with TD	Sujbects without TD	Crude OR	Adjuested OR
	(n=60)	(n=493)	(95% CI)	(95% CI)
MAFLD (-)	25 (6.4)	367 (93.6)	Ref	Ref
MAFLD (+)	35 (21.7)	126 (78.3)	4.08 (2.35-7.08)	4.74 (2.54-8.84)
(B)Fatty liver index≧60				
	Subjects with TT<300ng/dl	Sujbects with TT≧300 ng/dl	Crude OR	Adjuested OR^*^
	(n=68)	(n=485)	(95% CI)	(95% CI)
MAFLD (-)	56 (11.2)	445 (88.8)	Ref	Ref
MAFLD (+)	12 (23.1)	40 (76.9)	2.38 (1.18-4.81)	3.02 (1.29-7.06)
	Subjects with TD	Sujbects without TD	Crude OR	Adjuested OR
	(n=60)	(n=493)	(95% CI)	(95% CI)
MAFLD (-)	48 (9.6)	453 (90.4)	tpRef	Ref
MAFLD (+)	12 (23.1)	40 (76.9)	2.83 (1.39-5.76)	3.41 (1.45-8.05)

MAFLD, metabolic dysfunction associated fatty liver disease; TT, total testosterone; TD, testosterone deficiency; OR, odds ratio; CI, confidence interval.

^*^Adjusted for age, education status, and habits of smoking, alcohol drinking, betel quid chewing and regular exercise.

Fatty liver, in addition to its adverse effects on liver health, has been found to be closely associated with MetS and its individual components, establishing it as the hepatic manifestation of MetS (8). The presence of fatty liver disrupts normal intrahepatic glucose and triglyceride metabolism, leading to aggravated insulin resistance and lipid abnormalities, which in turn contribute to the progression of MetS (16, 27). Recent large-scale meta-analyses of prospective studies by Ballestri et al. and Mantovani et al. have also demonstrated that both enzyme- and ultrasound-diagnosed NAFLD significantly increase the risk of future development of MetS and type 2 diabetes in affected individuals (17, 28). These findings highlight the bidirectional relationship between fatty liver and MetS, emphasizing the crucial role of fatty liver in the pathogenesis and future risk of metabolic disorders.

Due to several limitations of the traditional definition of NAFLD, an international expert panel proposed the adoption of a new term, MAFLD, to replace the term NAFLD in 2020 (12, 13). This new term aims to emphasize the importance of metabolic dysfunction in the pathogenesis and progression of liver disease (12, 13). The recommendation for the use of MAFLD has gained support from over 1,000 important medical, healthcare, and pharmaceutical organizations across 134 countries as of 2022 (29). Recent studies have also demonstrated that the use of MAFLD diagnosis helps identify patients with a higher risk of metabolic dysfunction, liver-related complications, and other comorbidities (such as cardiovascular disease, chronic kidney disease, and colorectal adenomas) (11, 30). Therefore, the transition to the MAFLD diagnosis is considered a significant milestone, promoting a more inclusive, equitable, and patient-centered approach to addressing the significant challenges posed by fatty liver disease (11, 30).

In men, the impact of MetS on sexual and reproductive function has been extensively studied, while the role of fatty liver in this context has gained recognition only in recent years (31), particularly regarding its potential association with testosterone deficiency (32). As the liver is responsible for the metabolism of endogenous hormones and the production of the SHBG, the occurrence of fatty liver may disrupt hormone metabolism and SHBG production. Furthermore, it can potentially affect the hypothalamic-pituitary-gonadal axis by triggering insulin resistance, oxidative stress, and chronic inflammation, ultimately leading to decreased T levels within the body (31).

Recently, Jaruvongvanich et al. conducted a meta-analysis of 16 studies involving 13,721 males and 5,840 females. They found that males with NAFLD had approximately 2.78 nmol/L lower levels of TT compared to those without NAFLD (15). In another study by Phan et al., analyzing data from the National Health and Nutrition Examination Survey (NHANES), they also found that individuals with NAFLD diagnosed by ultrasound had significantly lower levels of TT and SHBG compared to those without NAFLD (33). In line with these findings, our previous study also found that FLI, a non-invasive screening tool for NAFLD, is significantly associated with the risk of TD in aging men (21). Together, these studies underscore the significant association between fatty liver and testosterone levels in men.

To date, there is limited research on the association between MAFLD and TD. Only one study by Cao et al. in 2022 has investigated this relationship in a cohort of 732 individuals (304 males and 428 females) from a Shanghai community. The found that males with MAFLD exhibited significantly diminished levels of TT and SHBG in comparison to their counterparts without MAFLD (both p<0.01) (18). Our findings are consistent with this study, providing additional evidence supporting their observations (**Table 2**). Additionally, among individuals without MetS, a population traditionally considered to be at lower risk of TD, those with MAFLD exhibited a 3.89-fold increased risk of having TT levels below 300 ng/dL and a 4.74-fold increased risk of having TD compared to those without MAFLD (**Table 4**). These results emphasize the potential influence of MAFLD on testosterone levels and the heightened risk of TD, particularly in individuals without MetS. Furthermore, the identification of MAFLD as an early predictor for TD in clinical practice can assist healthcare professionals in identifying individuals at high risk and implementing timely preventive interventions and treatment strategies in clinical settings.

There is evidence that low T levels are associated with disruptions in energy metabolism, alterations in body composition, increased accumulation of visceral fat, and the development of insulin resistance (32, 34). Testosterone therapy has been shown to be effective in alleviating symptoms associated with TD and offers additional benefits. It can enhance muscle mass, improve bone density, reduce body fat, and lower total cholesterol levels physiologically (35, 36). Furthermore, in individuals with TD who also have MetS or diabetes, a comprehensive meta-analysis conducted by Li et al. in 2020, comprising 18 randomized controlled trials, revealed significant improvements in blood glucose control (glycosylated hemoglobin), insulin sensitivity (HOMA index), lipid abnormalities (low-density lipoprotein and TGs), and body composition (weight, BMI, and WC) following testosterone therapy (37). Recent studies have also suggested that testosterone therapy may have positive effects on improving hepatic steatosis in individuals with TD and fatty liver (38, 39). These findings underscore the reciprocal relationship between TD and fatty liver, the potential therapeutic benefits of testosterone therapy extending beyond symptom relief and suggest its potential role in managing the metabolic and hepatic complications associated with TD, including MAFLD. Further research is essential to elucidate the underlying mechanisms and clinical implications of this association.

This study has several limitations. Firstly, the data were obtained from a community-based health screening program, potentially introducing selection bias despite its open participation. Therefore, larger population-based studies are needed to address this bias. Secondly, the findings relied on a single measurement of serum T level, although efforts were made to minimize diurnal variation. However, a single measurement may not fully reflect the actual T levels of the participants. Thirdly, due to the cross-sectional nature of the study, causality between MAFLD and TD could not be established. Finally, imaging techniques such as ultrasonography or liver histology were not performed to confirm the presence of fatty liver. Further research is required to evaluate the differences and compare the effectiveness of using the FLI for diagnosing MAFLD with the results obtained from imaging or pathological studies.

## 5 Conclusion

In our study of aging Taiwanese men, individuals with MAFLD exhibited significantly elevated rates of TT levels below 300 ng/dL, TD, and MetS compared to those without MAFLD. Notably, among individuals without MetS, a population traditionally considered to be at lower risk for TD, the presence of MAFLD was associated with a substantial increase in the risk of TT levels below 300 ng/dL and TD. These findings underscore the considerable impact of MAFLD on T levels and the heightened susceptibility to TD, particularly among individuals without MetS. Recognition of MAFLD as an early predictor of TD in clinical practice can facilitate the identification of high-risk individuals and the implementation of timely preventive interventions and therapeutic strategies. Further investigations involving diverse age and ethnic populations are necessary to validate our preliminary findings and unravel the underlying mechanisms that connect MAFLD, TD, and MetS.

## Data availability statement

The original contributions presented in the study are included in the article/**Supplementary Material**. Further inquiries can be directed to the corresponding author.

## Ethics statement

The studies involving humans were approved by The Institutional Research Ethics Committee of the Kaohsiung Medical University Hospital. The studies were conducted in accordance with the local legislation and institutional requirements. The participants provided their written informed consent to participate in this study.

## Author contributions

Research design: C-CL, S-PH, Y-CL, and T-JH. The acquisition of data: C-CL, S-PH, Y-CL, C-HL, T-YH, J-HG, C-WC, C-YL, Y-SJ, and W-JW. The analysis and interpretation of data: C-CL and T-JH. Drafting the paper: C-CL. Revising the paper critically: C-CL and T-JH; Approval of the submitted and final versions: C-CL, S-PH, Y-CL, and T-JH. All authors contributed to the article and approved the submitted version.

## References

[1] Liu CC Wu WJ Lee YC Wang CJ Ke HL Li WM. The prevalence of and risk factors for androgen deficiency in aging Taiwanese men. J Sex Med (2009) 6(4):936–46. doi: 10.1111/j.1743-6109.2008.01171.x 19210712

[2] Khera M Broderick GA Carson CC 3rd Dobs AS Faraday MM Goldstein I. Adult-onset hypogonadism. Mayo Clin Proc (2016) 91(7):908–26. doi: 10.1016/j.mayocp.2016.04.022 27343020

[3] Ciardullo S Zerbini F Cannistraci R Muraca E Perra S Oltolini A. Differential association of sex hormones with metabolic parameters and body composition in men and women from the United States. J Clin Med (2023) 12(14):4783. doi: 10.3390/jcm12144783 37510898 PMC10381414

[4] Kloner RA Carson C 3rd Dobs A Kopecky S Mohler ER 3rd. Testosterone and cardiovascular disease. J Am Coll Cardiol (2016) 67(5):545–57. doi: 10.1016/j.jacc.2015.12.005 26846952

[5] Corona G Rastrelli G Di Pasquale G Sforza A Mannucci E Maggi M. Endogenous testosterone levels and cardiovascular risk: meta-analysis of observational studies. J Sex Med (2018) 15(9):1260–71. doi: 10.1016/j.jsxm.2018.06.012 30145097

[6] Mulhall JP Trost LW Brannigan RE Kurtz EG Redmon JB Chiles KA. Evaluation and management of testosterone deficiency: AUA guideline. J Urol (2018) 200(2):423–32. doi: 10.1016/j.juro.2018.03.115 29601923

[7] Younossi ZM Koenig AB Abdelatif D Fazel Y Henry L Wymer M. Global epidemiology of nonalcoholic fatty liver disease-Meta-analytic assessment of prevalence, incidence, and outcomes. Hepatology (2016) 64(1):73–84. doi: 10.1002/hep.28431 26707365

[8] Powell EE Wong VW Rinella M. Non-alcoholic fatty liver disease. Lancet (2021) 397(10290):2212–24. doi: 10.1016/S0140-6736(20)32511-3 33894145

[9] Polyzos SA Kountouras J Mantzoros CS. Obesity and nonalcoholic fatty liver disease: From pathophysiology to therapeutics. Metabolism (2019) 92:82–97. doi: 10.1016/j.metabol.2018.11.014 30502373

[10] Younossi ZM Golabi P de Avila L Paik JM Srishord M Fukui N. The global epidemiology of NAFLD and NASH in patients with type 2 diabetes: A systematic review and meta-analysis. J Hepatol (2019) 71(4):793–801. doi: 10.1016/j.jhep.2019.06.021 31279902

[11] Alharthi J Gastaldelli A Cua IH Ghazinian H Eslam M. Metabolic dysfunction-associated fatty liver disease: a year in review. Curr Opin Gastroenterol (2022) 38(3):251–60. doi: 10.1097/MOG.0000000000000823 35143431

[12] Eslam M Newsome PN Sarin SK Anstee QM Targher G Romero-Gomez M. A new definition for metabolic dysfunction-associated fatty liver disease: An international expert consensus statement. J Hepatol (2020) 73(1):202–9. doi: 10.1016/j.jhep.2020.03.039 32278004

[13] Eslam M Sanyal AJ George J. MAFLD: A consensus-driven proposed nomenclature for metabolic associated fatty liver disease. Gastroenterology (2020) 158(7):1999–2014.e1. doi: 10.1053/j.gastro.2019.11.312 32044314

[14] Mintziori G Poulakos P Tsametis C Goulis DG. Hypogonadism and non-alcoholic fatty liver disease. Minerva Endocrinol (2017) 42(2):145–50. doi: 10.23736/S0391-1977.16.02570-0 27879963

[15] Jaruvongvanich V Sanguankeo A Riangwiwat T Upala S. Testosterone, sex hormone-binding globulin and nonalcoholic fatty liver disease: a systematic review and meta-analysis. Ann Hepatol (2017) 16(3):382–94. doi: 10.5604/01.3001.0009.8593 28425408

[16] Lonardo A Ballestri S Marchesini G Angulo P Loria P. Nonalcoholic fatty liver disease: a precursor of the metabolic syndrome. Dig Liver Dis (2015) 47(3):181–90. doi: 10.1016/j.dld.2014.09.020 25739820

[17] Ballestri S Zona S Targher G Romagnoli D Baldelli E Nascimbeni F. Nonalcoholic fatty liver disease is associated with an almost twofold increased risk of incident type 2 diabetes and metabolic syndrome. Evidence from a systematic review and meta-analysis. J Gastroenterol Hepatol (2016) 31(5):936–44. doi: 10.1111/jgh.13264 26667191

[18] Cao W Xu Y Shen Y Wang Y Ma X Bao Y. Associations between sex hormones and metabolic-associated fatty liver disease in a middle-aged and elderly community. Endocr J (2022) 69(8):1007–14. doi: 10.1507/endocrj.EJ21-0559 35321990

[19] Liu CC Huang SP Cheng KH Hsieh TJ Huang CN Wang CJ. Lower SHBG level is associated with higher leptin and lower adiponectin levels as well as metabolic syndrome, independent of testosterone. Sci Rep (2017) 7(1):2727. doi: 10.1038/s41598-017-03078-0 28577342 PMC5457423

[20] Liu CC Lee YC Huang SP Cheng KH Hsieh TJ Huang TY. Hepatocyte nuclear factor-4alpha P2 promoter variants are associated with the risk of metabolic syndrome and testosterone deficiency in aging Taiwanese men. J Sex Med (2018) 15(11):1527–36. doi: 10.1016/j.jsxm.2018.09.012 30415809

[21] Liu CC Huang SP Hsieh TJ Lee CH Cheng KH Huang TY. Fatty liver index is associated with the risk of testosterone deficiency in aging men without metabolic syndrome. Andrology (2021) 9(3):863–72. doi: 10.1111/andr.12979 33484089

[22] Chueh KS Huang SP Lee YC Wang CJ Yeh HC Li WM. The comparison of the aging male symptoms (AMS) scale and androgen deficiency in the aging male (ADAM) questionnaire to detect androgen deficiency in middle-aged men. J Androl (2012) 33(5):817–23. doi: 10.2164/jandrol.111.015628 22240559

[23] Liu CC Huang SP Wu WJ Chou YH Juo SH Tsai LY. The impact of cigarette smoking, alcohol drinking and betel quid chewing on the risk of calcium urolithiasis. Ann Epidemiol (2009) 19(8):539–45. doi: 10.1016/j.annepidem.2009.02.006 19375945

[24] Cheng KH Huang SP Huang CN Lee YC Chu CS Chang CF. The impact of estradiol and 1,25(OH)2D3 on metabolic syndrome in middle-aged Taiwanese males. PloS One (2013) 8(3):e60295. doi: 10.1371/journal.pone.0060295 23555948 PMC3610656

[25] Bedogni G Bellentani S Miglioli L Masutti F Passalacqua M Castiglione A. The Fatty Liver Index: a simple and accurate predictor of hepatic steatosis in the general population. BMC Gastroenterol (2006) 6:33. doi: 10.1186/1471-230X-6-33 17081293 PMC1636651

[26] Yang BL Wu WC Fang KC Wang YC Huo TI Huang YH. External validation of fatty liver index for identifying ultrasonographic fatty liver in a large-scale cross-sectional study in Taiwan. PloS One (2015) 10(3):e0120443. doi: 10.1371/journal.pone.0120443 25781622 PMC4363626

[27] Yki-Jarvinen H. Non-alcoholic fatty liver disease as a cause and a consequence of metabolic syndrome. Lancet Diabetes Endocrinol (2014) 2(11):901–10. doi: 10.1016/S2213-8587(14)70032-4 24731669

[28] Mantovani A Byrne CD Bonora E Targher G. Nonalcoholic fatty liver disease and risk of incident type 2 diabetes: A meta-analysis. Diabetes Care (2018) 41(2):372–82. doi: 10.2337/dc17-1902 29358469

[29] Méndez-Sánchez N Bugianesi E Gish RG Lammert F Tilg H Nguyen MH. Global multi-stakeholder endorsement of the MAFLD definition. Lancet Gastroenterol Hepatol (2022) 7(5):388–90. doi: 10.1016/S2468-1253(22)00062-0 35248211

[30] Kawaguchi T Tsutsumi T Nakano D Torimura T. MAFLD: Renovation of clinical practice and disease awareness of fatty liver. Hepatol Res (2022) 52(5):422–32. doi: 10.1111/hepr.13706 34472683

[31] Hawksworth DJ Burnett AL. Nonalcoholic fatty liver disease, male sexual dysfunction, and infertility: common links, common problems. Sex Med Rev (2020) 8(2):274–85. doi: 10.1016/j.sxmr.2019.01.002 30898592

[32] Hermoso DAM Bizerra PFV Constantin RP Ishii-Iwamoto EL Gilglioni EH. Association between metabolic syndrome, hepatic steatosis, and testosterone deficiency: evidences from studies with men and rodents. Aging Male (2020) 23(5):1296–315. doi: 10.1080/13685538.2020.1764927 32406295

[33] Phan H Richard A Lazo M Nelson WG Denmeade SR Groopman J. The association of sex steroid hormone concentrations with non-alcoholic fatty liver disease and liver enzymes in US men. Liver Int (2021) 41(2):300–10. doi: 10.1111/liv.14652 PMC10115140 32860311

[34] Mody A White D Kanwal F Garcia JM. Relevance of low testosterone to non-alcoholic fatty liver disease. Cardiovasc Endocrinol (2015) 4(3):83–9. doi: 10.1097/XCE.0000000000000057 PMC4577238 26405614

[35] Bhasin S Brito JP Cunningham GR Hayes FJ Hodis HN Matsumoto AM. Testosterone therapy in men with hypogonadism: an endocrine society clinical practice guideline. J Clin Endocrinol Metab (2018) 103(5):1715–44. doi: 10.1210/jc.2018-00229 29562364

[36] Fode M Salonia A Minhas S Burnett AL Shindel AW. Late-onset hypogonadism and testosterone therapy - A summary of guidelines from the american urological association and the european association of urology. Eur Urol Focus (2019) 5(4):539–44. doi: 10.1016/j.euf.2019.02.021 30858073

[37] Li SY Zhao YL Yang YF Wang X Nie M Wu XY. Metabolic effects of testosterone replacement therapy in patients with type 2 diabetes mellitus or metabolic syndrome: A meta-analysis. Int J Endocrinol (2020) 2020:4732021. doi: 10.1155/2020/4732021 33061966 PMC7545471

[38] Albhaisi S Kim K Baker J Chidambaram N Patel MV Charlton M. LPCN 1144 resolves NAFLD in hypogonadal males. Hepatol Commun (2020) 4(10):1430–40. doi: 10.1002/hep4.1571 PMC7527694 33024914

[39] Apostolov R Gianatti E Wong D Kutaiba N Gow P Grossmann M. Testosterone therapy reduces hepatic steatosis in men with type 2 diabetes and low serum testosterone concentrations. World J Hepatol (2022) 14(4):754–65. doi: 10.4254/wjh.v14.i4.754 PMC9099110 35646271

