# Supplementary material for: Metabolic dysfunction-associated fatty liver disease is an early predictor for testosterone deficiency in aging men without metabolic syndrome
Source: Front Endocrinol (Lausanne). 2023 Oct 3;14:1252774. doi: 10.3389/fendo.2023.1252774 (PMC10579790; doi:10.3389/fendo.2023.1252774)
Supplement: Supplementary file 1 [file Table_1.docx]

**Supplementary Material**

**Table S1** **Selection criteria for study population**

| Inclusion criteria | Taiwanese males aged≧40 years |
| --- | --- |
| Exclusion criteria | Significant psychiatric disorders |
|  | Substance abuse disorders |
|  | Advanced liver disease |
|  | Advanced renal disease |
|  | Malignancy |
|  | Current hormone therapy |
|  | Current anti-androgen treatment |
|  | Current steroidal agent treatment |
|  | Current antifungal drug treatment |
|  | Incomplete evaluation |
